# The impact of different anti-vascular endothelial growth factor treatment regimens on reducing burden for caregivers and patients with wet age-related macular degeneration in a single-center real-world Japanese setting

**DOI:** 10.1371/journal.pone.0189035

**Published:** 2017-12-08

**Authors:** Tsukasa Hanemoto, Yusuke Hikichi, Norimasa Kikuchi, Tadahiko Kozawa

**Affiliations:** 1 Department of Ophthalmology, Kozawa Eye Hospital, Ibaraki, Japan; 2 Market Access, Health Economics and Outcomes Research, Bayer Yakuhin, Ltd., Tokyo, Japan; 3 CR Division, Clinical Study Support, Inc., Nagoya, Japan; University of Utah (Salt Lake City), UNITED STATES

## Abstract

**Objective:**

To describe the burden associated with different anti-vascular endothelial growth factor (VEGF) treatment strategies for wet age-related macular degeneration (wAMD) in a real-word setting in Japan.

**Methods:**

Single-center, cross-sectional survey of caregivers of patients with wAMD performed in a hospital in Mito-City, a rural area in Japan. Caregiver burden was evaluated using the Burden Index of Caregivers (BIC-11), and depressive symptoms were assessed by the Center for Epidemiologic Studies Depression scale. Retrospective medical chart review was conducted to monitor resource use and visual acuity outcomes in patients. The productivity loss of caregivers accompanying patients on hospital visits was estimated using the human capital method.

**Results:**

Seventy-one patient-caregiver pairs were included. Most caregivers were female (74.6%), spouse/partner (54.9%), employed (46.5%), and the primary caregiver (85.9%). Patients received anti-VEGF treatment as follows: treat-and-extend (T&E; n = 42), switch (from as-needed [PRN] to T&E; n = 18), PRN (n = 10), and other (n = 1). Caregiver-related burden (total BIC-11 scores) were 4.29 (T&E) 4.60 (PRN), and 5.33 (switch) (p = NS).

The mean number of hospital visits was lower with T&E than PRN (7.88 vs. 14.0 [p = 0.00674] in year 1 and 5.68 vs. 9.0 in year 2). For patients who switched from PRN to T&E, the mean number of hospital visits decreased from 13.21 to 7.43 (p<0.0001) in the first year after switch. The productivity loss associated with accompanying patients to the hospital was lower for caregivers of patients receiving T&E than PRN (mean differences: 74,456.04 JPY [p = 0.00284] in year 1 and 40843.14 JPY in year 2), and was also reduced for caregivers of patients who switched from PRN to T&E.

**Conclusion:**

wAMD treatment with anti-VEGF agents via T&E reduced hospital visits compared with PRN, where associated monitoring visits are necessary to provide good patient outcomes. T&E was associated with a reduction trend in caregiver burden, including time and costs.

## Introduction

It is estimated that nearly 200,000 adults in Japan have visual impairment and reduced quality of life associated with age-related macular degeneration (AMD) [1]. The Hisayama study showed that the 9-year cumulative incidences of early and late AMD were 10% and 1.4%, respectively, which increased with advancing age [2]; it is likely that this number will increase dramatically with an aging demographic in Asia [3].

Anti-vascular endothelial growth factor (VEGF) agents are effective and well tolerated in the treatment of wet AMD (wAMD) [4–8]. However, wAMD still places an enormous burden on patients and their caregivers. A number of surveys have shown that the emotional, time-related, and financial impact of caring for patients with wAMD on non-paid caregivers may be underestimated [9–16]. Based on findings from validated questionnaires, the burden of caregiving for patients with wAMD may be equivalent to that experienced by caregivers for patients with multiple sclerosis [12,17], and the level of depression is comparable to that experienced by the wAMD patients themselves [10]. It is therefore important to identify ways to reduce this caregiver burden. A previous qualitative study performed at the Kozawa Eye Hospital in Japan found that caregiver burden was associated with the number of hospital visits [18]. Similar outcomes have been observed in the United Kingdom; in this study of 250 matched patient-caregiver pairs, the most common caregiver activity was accompanying patients to clinic visits, with 43.6% of caregivers spending most of the day at the clinic every 4–6 weeks [12].

In light of these findings, a further study was performed at the Kozawa Eye Hospital to determine how different anti-VEGF treatment regimens could reduce caregiver burden and hospital visits. Of particular interest was the impact that anti-VEGF treat-and-extend (T&E) regimens could have in reducing this burden compared with as-needed (PRN) regimens. T&E has been shown to reduce injection/visit frequency compared with monthly regimens while maintaining visual acuity outcomes in patients with wAMD [19]. Outcomes were also maintained when patients with wAMD were switched from intravitreal aflibercept (IVT-AFL) PRN to T&E [20,21]. This paper reports the findings from the Kozawa Eye Hospital study in Japan.

## Materials and methods

### Study design

This was a single-center, observational study (ClinicalTrials.gov identifier: NCT02541084) consisting of a cross-sectional survey of caregivers of patients with wAMD, and a retrospective medical chart review of patients with wAMD in Japan. Participants were recruited during visits to the Kozawa Eye Hospital between 18 August 2015 and 31 March 2016. Kozawa Eye Hospital is located in a rural area called Mito-City, Ibaraki Prefecture. The population is approximately 270,000. Public transportation is limited, and thus car dependence is very high. The observational study was conducted in accordance with the ethical guidelines for epidemiologic studies (2015) [22] and the Declaration of Helsinki. The study also complied with all local laws, and ethical committee approval was obtained from Kozawa Eye Hospital. Written informed consent was collected from each patient and caregiver prior to inclusion in the study.

### Objectives

The primary aim was to explore the degree of burden for caregivers of patients with wAMD being treated with anti-VEGF therapy (IVT-AFL or ranibizumab T&E vs. PRN regimens). The study also monitored the resource use and visual acuity outcomes in patients with wAMD who were previously naïve or had switched from an anti-VEGF (IVT-AFL or ranibizumab) PRN regimen to T&E.

### Participants

Patient-caregiver pairs who had attended hospital visits for wAMD monitoring and treatment were recruited. Patients were also required to be diagnosed with wAMD and treated with anti-VEGF therapy PRN or T&E for ≥1 year prior to study inclusion. Patients could be previously naïve, or previously treated with IVT-AFL, ranibizumab, pegaptanib sodium PRN, or photodynamic therapy. Patients were excluded if they had a history of intraocular surgery for other eye diseases after the initiation of wAMD treatment or if they had been previously treated with bevacizumab for wAMD. Caregivers were required to be non-professionals (i.e., non-paid) who accompanied patients on hospital visits and were capable of understanding and completing the questionnaire without assistance.

### Data collection

For caregivers, the baseline characteristics, demographics, and caregiving burden were identified using self-administered questionnaires, including the Burden Index of Caregivers-11 (BIC-11) and the Center for Epidemiologic Studies Depression Scale (CES-D). The BIC-11 and CES-D have both been validated in English and Japanese [23–25]. The BIC-11 is a measure of caregiver burden in five domains (time-dependent, emotional, existential [spiritual], physical, and service-related). Caregivers self-assess 11 items in each domain using five-point Likert scales (from 0 = never to 4 = always). The BIC-11 was validated in Japan using feedback from caregivers of patients with neurological disorders. The total score ranged from 0 to 44, with a higher score indicating greater caregiver burden; no cut-off was identified. The CES-D measures depressive mood; each caregiver self-assesses the number of days per week that they are affected by depression from “none” to “≥5 days” based on 20 symptomatic items, providing a total score ranging from 0 to 60. A higher score indicates greater depressive mood, and the cut-off for depression was a score ≥16 in Japanese patients.

Patient demographics and medical history were collected retrospectively through a chart review conducted by the investigator and study coordinators. No additional treatments or examinations were conducted to obtain information required for this study; only existing available medical records were used. No safety data were reported. To ensure accuracy and reliability of the data, questionnaire responses were entered into an electronic data capture system by a study coordinator and were independently reviewed by another study coordinator to verify that the data had been handled appropriately and accurately.

### Caregiver assessments

The primary caregiver assessments included caregiver burden measured by the BIC-11, and the time and financial burden of caregiving and for hospital visits. Secondary caregiver assessments included depressive symptoms assessed using the CES-D. Caregivers of patients assigned to each treatment group (PRN, T&E, and PRN to T&E switchers) completed the questionnaires once at screening after informed consent from patients and caregivers had been obtained.

### Patient treatment and assessments

The investigators conducted a chart review to understand previous treatment patterns including frequency of visits, tests, and injections for each patient, and based on the data, the investigators decided which treatment group each patient should be assigned to (PRN or T&E). The PRN regimen comprised a loading phase (three injections within 90 days), followed by frequent monitoring (nearly every month) and treatment if needed based on visual and anatomic tests. The predefined T&E regimen comprised a loading phase (three injections within 90 days) followed by treatment every 2–3 months depending on patient disease conditions; the treatments could be extended up to 4 months if disease stability was confirmed, or shortened to 2 months if subretinal fluid or edema was observed.

The secondary patient-related assessments included resource use (number of hospital visits and injections) and visual acuity outcomes (mean change in best-corrected visual acuity [BCVA], Early Treatment Diabetic Retinopathy Study [ETDRS] letters) over a 2 year period after the start of anti-VEGF therapy (PRN or T&E), including a comparison before and after switch from PRN to T&E. The switch group included patients who changed from anti-VEGF PRN to T&E, and assessments were made in patients who had 1 and 2 years of follow-up for both regimens.

### Statistical analysis

A total of 200 patient-caregiver pairs were intended to be enrolled based on the number of eligible participants estimated by the principal investigator at the hospital. As the study was exploratory, no power analysis for the sample size calculation was planned.

Continuous data were described by the median, mean and standard deviation (SD). Categorical data were reported using frequencies and percentages. For the BIC-11 and the CES-D, item-level missing data were handled in accordance with scoring documentation provided for each instrument. In the absence of missing data, the 50% rule was used, i.e., domains with ≥50% non-missing-item responses were scored as the average of the non-missing responses (with reverse scoring and rescaling as appropriate). Missing data in other items in the caregiver questionnaire were not replaced. If any other data were not available at the participating site (e.g., baseline characteristics), then only the available information was reported.

The baseline characteristics and demographics of patient-caregiver pairs were described using summary statistics. The primary caregiver assessments (BIC-11 total and domain scores) were compared between treatment categories using *t* tests and between visual acuity categories using trend tests (due to small numbers) at the 5% (p<0.05) significance level. The effects of preselected caregiver and patient variables (such as age and sex) on BIC-11 scores were assessed using analysis of variance or Pearson’s correlation coefficient. The costs of accompanying patients to hospital by treatment category were analyzed using the Human Capital Method. The secondary caregiver assessments (CES-D scores) and secondary patient assessments were compared between treatment categories and preselected variables (such as age, sex, and visual acuity) using summary statistics to understand numerical or distribution trends only with p-values as secondary (subsidiary) data.

For cost analyses, caregiving burden was defined as the entire time and cost spent taking care of wAMD patients, and hospital visits included the time and productivity loss associated with accompanying patients on hospital visits. The productivity loss of caregivers was estimated using human capital method. The annual productivity loss for accompanying patients on hospital visits was determined using the following formula: ([estimated daily wage + travel cost per visit] × number of visits per year). Daily wage was estimated based on the basic survey on wage structure (2015) reported by the Ministry of Health, Labour and Welfare of Japan (2015) [26]. The daily wage of non-employed caregivers was calculated according to the method devised by Drummond et al. (2001) (i.e., non-paid working time should be valued at the net wage that could be obtained by the individual if they were in paid employement) [27]. The statistical analyses were performed using summary statistics in SAS for Windows, version 9.2 or higher (SAS Institute Inc., Cary, NC, USA).

## Results

### Participants

**Fig 1:**
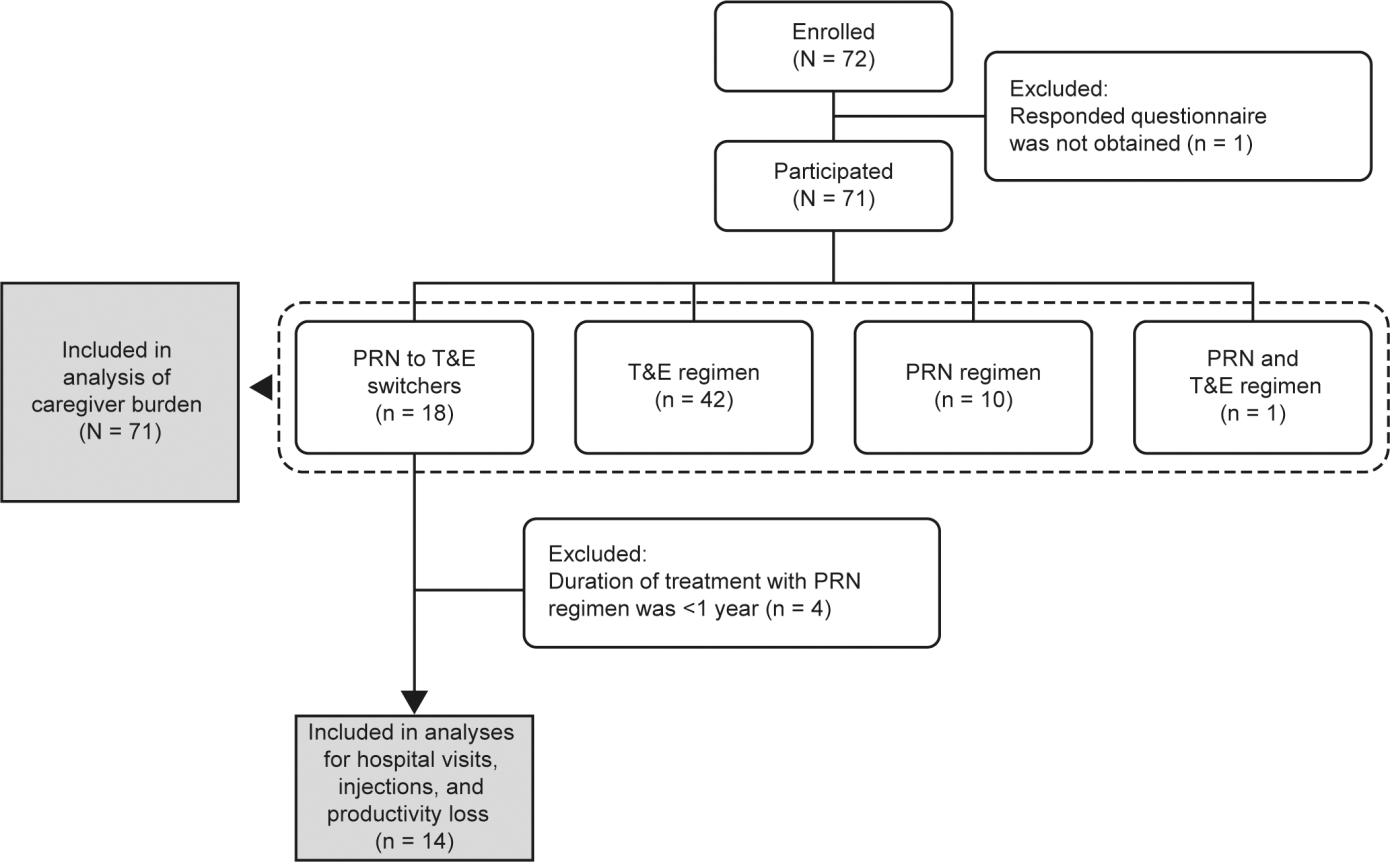
Disposition of participants. PRN, as-needed; T&E, treat-and-extend.

A total of 72 patient-caregiver pairs were enrolled (Fig 1). As one caregiver did not respond, 71 pairs were included in the caregiver analyses. This was lower than the original estimate (200 pairs), which was mainly due to overestimation of patients meeting the inclusion criteria, as well as other patient considerations, including treatment costs. Patient treatment categories were T&E (n = 42), PRN to T&E switch (n = 18), PRN (n = 10), and other (n = 1). Nearly all patients (89%) had received the T&E regimen for ≥1 year prior to study inclusion. The baseline characteristics of the patient-caregiver pairs (N = 71) are presented in Table 1 [28]. For caregivers, the mean (SD) age was 63.9 (12.7) years and 74.6% were female. Most caregivers were a spouse/partner (54.9%) or child (38.0%) of the patient, and lived with the patient (67.6%). A total of 33 caregivers (46.5%) were employed (Table 1A). For patients (N = 71), the mean (SD) age was 77.9 (8.0) years and 71.8% were male. Nineteen patients (26.8%) had bilateral wAMD. The mean (SD) duration of disease was 47.06 (33.03) months and 45.1% had comorbidities, with solid tumors (14.1%) and peptic ulcer disease (11.3%) being the most common (Table 1B).

**Table 1:** Baseline characteristics and demographics of caregivers and patients.

**A. Caregivers**	**N = 71**
Female, n (%)	53 (74.6)
Age, years, mean (SD)	63.9 (12.7)
Relationship with patient, n (%)	
Spouse/partner	39 (54.9)
Son/daughter (including son- or daughter-in-law)	27 (38.0)
Grandson/granddaughter	2 (2.8)
Brother/sister (including brother- or sister-in-law)	3 (4.2)
Living with patient, n (%)	48 (67.6)
Educational background, n (%)	
Junior high school	10 (14.1)
High school	33 (46.5)
Junior college/vocational school	19 (26.8)
College	9 (12.7)
Employment status, n (%)	
Employed full-time	15 (21.1)
Employed part-time	10 (14.1)
Self-employed	8 (11.3)
Full-time housekeeper	25 (35.2)
Not employed (including retired or pensioner)	13 (18.3)
Annual household income, JPY, n (%)	
<2,500,000	13 (18.3)
2,500,000–4,999,999	27 (38.0)
5,000,000–7,499,999	12 (16.9)
7,500,000–9,999,999	4 (5.6)
10,000,000–12,499,999	3 (4.2)
12,500,000–14,999,999	0 (0.0)
≥15,000,000	2 (2.8)
Decline to answer	10 (14.1)
**B. Patients**	**N = 71**
Female, n (%)	20 (28.2)
Age, years, mean (SD)	77.9 (8.0)
Treating eye, n (%)	
Unilateral	52 (73.2)
Bilateral	19 (26.8)
wAMD subtype (90 eyes), n (%)	
tAMD	49 (54.4)
PCV	29 (32.2)
RAP	12 (13.3)
Disease duration, months (90 eyes), mean (SD)	47.06 (33.03)
Comorbidities, n (%)	
Myocardial infarction	3 (4.2)
Peripheral vascular disease	3 (4.2)
Cerebrovascular disease	5 (7.0)
Dementia	1 (1.4)
Chronic obstructive pulmonary disease	3 (4.2)
Peptic ulcer disease	8 (11.3)
Diabetes mellitus	7 (9.9)
Solid tumor	10 (14.1)
Liver disease	1 (1.4)
Number of comorbidities per patient, n (%)	
One	23 (32.4)
Two	9 (12.7)
BCVA score (ETDRS letters) in better-seeing eye, mean (SD)	
PRN to T&E switchers [n = 18]	68.44 (14.79)
T&E [n = 39]	66.46 (16.45)
PRN [n = 10]	77.20 (11.88)
BCVA category (ETDRS letters) in better-seeing eye, n (%) [n = 67]*	
Blindness (<20 letters)	4 (5.97)
Severe visual impairment (≥20–<35 letters)	0
Moderate visual impairment (≥35–<58 letters)	3 (4.48)
Some visual impairment (≥58–<70 letters)	9 (13.43)
Mild visual impairment (≥70–<80 letters)	10 (14.93)
No visual impairment (≥80 letters)	41 (61.19)

BCVA, best-corrected visual acuity; ETDRS, Early Treatment Diabetic Retinopathy Study; JPY, Japanese Yen; PCV, polypoidal choroidal vasculopathy; PRN, as-needed; RAP, retinal angiomatous proliferation; SD, standard deviation; tAMD, typical age-related macular degeneration; T&E, treat-and-extend; wAMD, wet age-related macular degeneration.

*Visual impairment is defined according to World Health Organization categories (WHO 2016) [28].

### Primary caregiver assessments

#### Caregiver burden measured by the BIC-11

The mean (SD) total and domain BIC-11 scores for caregivers are shown in Table 2 [28], and the BIC-11 item response distribution is summarized in Table A in S1 File. The mean (SD) total score was 4.29 (5.49) for caregivers of patients using T&E, 4.60 (4.74) for caregivers of patients using PRN, and 5.33 (9.40) for caregivers of patients who switched from PRN to T&E (p = NS). Caregivers of patients who switched had a lower time-dependent burden.

**Table 2:** Total and domain BIC-11 scores for caregivers by patient treatment and visual impairment (better-seeing eye) categories.

	Domain, Mean (SD)						
	Time-dependent burden	Emotional burden	Existential burden	Physical burden	Service-related burden	Personal estimate of overall burden	Total score
Treatment category*							
PRN to T&E switchers (n = 18)	1.17 (2.23)	1.00 (1.81)	1.22 (1.96)	0.61 (1.69)	0.72 (1.74)	0.61 (0.98)	5.33 (9.40)
T&E (n = 42)	1.21 (1.59)	0.79 (1.22)	0.81 (1.31)	0.60 (0.99)	0.40 (0.70)	0.48 (0.63)	4.29 (5.49)
PRN (n = 10)	1.40 (0.97)	0.80 (1.03)	0.80 (1.03)	0.40 (0.84)	0.80 (1.03)	0.40 (0.70)	4.60 (4.74)
Patient’s actual visual impairment category^1^**							
Blindness (<20 letters) (n = 4)	2.00 (2.31)	1.25 (1.89)	2.00 (1.63)	1.00 (1.15)	1.00 (1.15)	0.75 (0.50)	8.00 (7.87)
Severe visual impairment (≥20–<35 letters) (n = 0)	—	—	—	—	—	—	—
Moderate visual impairment (≥35–<58 letters) (n = 3)	0.67 (1.15)	0.67 (1.15)	0.67 (1.15)	0	0.67 (1.15)	0.67 (1.15)	3.33 (5.77)
Some visual impairment (≥58–<70 letters) (n = 9)	1.78 (1.92)	1.22 (1.79)	0.89 (1.45)	0.67 (1.00)	0.56 (0.73)	0.67 (0.71)	5.78 (6.61)
Mild visual impairment (≥70–<80 letters) (n = 10)	2.10 (2.69)	1.60 (2.22)	1.80 (2.57)	0.90 (2.23)	1.30 (2.21)	0.80 (1.14)	8.50 (11.76)
No visual impairment (≥80 letters) (n = 41)	0.90 (1.24)	0.56 (0.87)	0.63 (1.02)	0.44 (0.90)	0.29 (0.64)	0.34 (0.62)	3.17 (4.30)
Caregiver’s rating of patient’s visual acuity**							
Mild (n = 26)	1.15 (1.59)	0.77 (0.99)	0.81 (1.20)	0.42 (0.90)	0.38 (0.75)	0.35 (0.63)	3.88 (4.91)
Moderate (n = 35)	1.09 (1.46)	0.69 (1.16)	0.71 (1.13)	0.49 (0.89)	0.46 (0.78)	0.54 (0.82)	3.97 (5.14)
Severe (n = 10)	1.80 (2.53)	1.50 (2.42)	1.80 (2.57)	1.20 (2.20)	1.20 (2.20)	0.70 (0.67)	8.20 (12.10)

Domain score for “personal estimate of overall burden” ranges from 0 to 4. Scores for other domains ranges from 0 to 8. Total scores ranges from 0 to 44. Higher score indicates greater burden. BIC-11, Burden Index of Caregivers-11; PRN, as-needed; SD, standard deviation; T&E, treat-and-extend.

^1^Visual impairment is defined according to World Health Organization categories (WHO, 2016) [28].

*p = not significant between treatment groups (switcher vs. PRN or T&E, T&E vs. PRN) for total and subscale scores (unpaired *t* test).

**p = not significant for total and subscales scores and worsening visual impairment (trend test).

There were no obvious associations between total BIC-11 score and patient’s actual visual impairment (better-seeing eye); the mean (SD) total score was 8.00 (7.87) for caregivers of patients categorized as blind, 3.33 (5.77) for caregivers of patients with moderate visual impairment, and 8.50 (11.76) for caregivers of patients with mild visual impairment (Table 2). However, when data where stratified according to the caregiver’s rating of the patient’s visual acuity, the BIC-11 total score numerically increased with increasing severity; this was not statistically significant using a trend test (Table 2). Total BIC-11 score was numerically higher, but not statistically significant, for caregivers of patients with bilateral compared with unilateral wAMD (6.25 [3.97] vs. 3.86 [6.69]; p = 0.147). However, there were no clear associations between total BIC-11 score and age or sex (patients and caregivers), caregiver status (primary caregiver or not), perceived stress for waiting time in hospital, or the number of hospital visits in the previous year (Table B in S1 File). The Pearson’s correlation coefficient (95% confidence interval) for caregiver age and number of hospital visits in the previous year was 0.03 (–0.20 to 0.26) and –0.12 (–0.35 to 0.12), respectively.

#### Time and productivity loss of caregiving and accompanying patients on hospital visits

**Fig 2:**
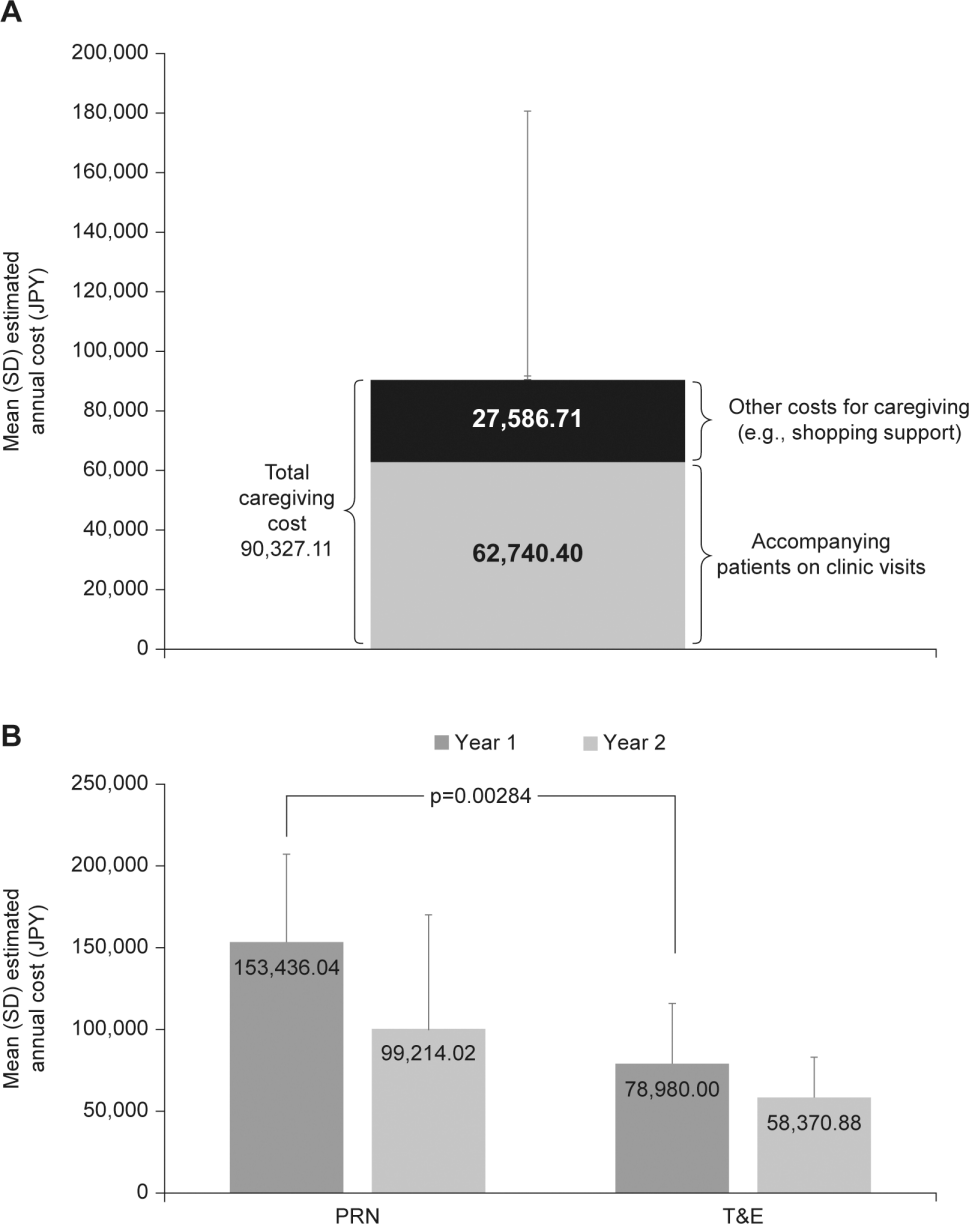
**Estimated annual productivity loss** for (A) caregiving and accompanying patients on hospital visits, and (B) by patient treatment category and treatment year in PRN and T&E groups. Total caregiving = entire cost for taking care of wAMD patients. PRN, as-needed; T&E, treat-and-extend; wAMD, wet age-related macular degeneration.

Most caregivers (85.9%) provided primary care (Table 3), and the mean (SD) time spent caregiving was 5.61 (7.7) hours per day. An additional analysis found that among the employed caregivers, the mean (SD) time absent from work due to caregiving was 1.18 (0.4) days each month. The estimated mean (SD) annual hours required for caregiving were 89.38 (196.18) for all caregivers, and 88.16 (210.34) hours for employed and 88.59 (182.22) hours for non-employed caregivers. The total annual cost associated with caregiving is shown in Fig 2A. Most of this cost was associated with accompanying patients on clinic visits.

**Table 3:** Caregiving-related information obtained via self-administered questionnaires.

	N = 71
Caregiver status (primary caregiver or not), n (%)	
Yes	61 (85.9)
No	10 (14.1)
Current use of a paid caregiving service, n (%)	
Yes	0 (0.0)
No	71 (100.0)
Frequency of accompanying patient to hospital visits, n (%)	
Every time, nearly every time (90–100% of the time)	57 (80.3)
Most of the time (75% of the time)	3 (4.2)
Nearly half the time (50% of the time)	3 (4.2)
Sometimes (25% of the time)	6 (8.5)
Rarely (10% or less of the time)	2 (2.8)
Transport expenses to the hospital per visit, JPY, mean (SD) (n = 13)	4546.76 (2846.2)
Distance to the hospital per visit, km, mean (SD) (n = 63)	62.6 (63.00)
Means of transportation, n (%)	
Private car	57 (80.3)
Taxi	3 (4.2)
Public transportation	3 (4.2)
Private car and public transportation	4 (5.6)
Public transportation and taxi	3 (4.2)
Private car and taxi	1 (1.4)
Remain in the hospital during patient’s medical examination, n (%)	
Yes	57 (80.3)
No	14 (19.7)
Perceived stress for waiting time, n (%) [n = 70]	
Very stressful	8 (11.4)
Moderately stressful	32 (45.7)
Slightly stressful	22 (31.4)
Not at all	8 (11.4)
Activity during waiting time in the hospital, n (%)*	
*In a hospital*	
Reading a book, magazine, etc	33 (46.5)
Watching TV in the hospital	35 (49.3)
Internet surfing (incl. e-mails) by smartphones, tablets, etc	10 (14.1)
Nothing in particular	20 (28.2)
*Not in a hospital*	
Shopping	10 (14.1)
Housework	9 (12.7)
Going back to work	4 (5.6)
Leisure	5 (7.0)
Other	1 (1.4)
Required caregiving in daily life, n (%)	13 (18.3)
Hours per day, mean (SD) (n = 9)	5.61 (7.7)
Caregiving required*	
Feeding	1 (1.4)
Grooming	1 (1.4)
Physical ambulation	3 (4.2)
Bathing	2 (2.8)
Toileting	1 (1.4)
Food preparation	4 (5.6)
Telephone usage	2 (2.8)
Shopping	10 (14.1)
Cleaning	3 (4.2)
Doing laundry	2 (2.8)
Responsibility for patient’s medication	4 (5.6)
Handling finance	5 (7.0)
Use of long-term government care insurance	7 (9.9)
Degree of vision problems (caregiver perspective)	
Low	26 (36.6)
Moderate	35 (49.3)
High	10 (14.1)

JPY, Japanese Yen; SD, standard deviation; wAMD, wet age-related macular degeneration.

*Multiple answers possible.

Most caregivers (80.3%) attended 90–100% of hospital visits. The majority of caregivers (80.3%) also stayed in the hospital during the patient’s visit. The mean (SD) duration per visit was 236.47 (48.5) minutes, and the mean (SD) annual time spent accompanying patients on hospital visits was 32.84 (13.73) hours. Many caregivers (57.1%; n = 40/70) rated the hospital waiting time as “moderately to very stressful.” The productivity loss associated with accompanying patients to hospital was lower for caregivers of patients receiving T&E than PRN; the mean differences were 74,456.04 JPY (p = 0.00284) in year 1 and 40843.14 JPY in year 2, corresponding to €616.60 and €338.22, respectively (Fig 2B). In patients who switched from PRN to T&E, the mean differences were 51,048.82 JPY (p<0.0001) in year 1 and 14,305.80 JPY in year 2, corresponding to €422.73 and €118.46, respectively (April 2017 Euro values [www.xe.com]). There was no obvious association between costs associated with hospital visits and the degree of patient visual impairment (Table 4) [28].

**Table 4:** Estimated annual costs for accompanying patients on hospital visits, and total annual caregiving cost by visual impairment category (better-seeing eye).

Impairment level* (ETDRS letters)	n	Estimated annual cost for accompanying patients on hospital visits, JPY	n	Estimated total annual caregiving cost, JPY
		Mean (SD)		Mean (SD)
Blindness (<20 letters)	0	–	0	–
Severe visual impairment (≥20–<35 letters)	1	76,645.91 (–)	1	76,645.91 (–)
Moderate visual impairment (≥35–<58 letters)	11	69,353.69 (30,146.58)	12	137,049.22 (159,013.80)
Some visual impairment (≥58–<70 letters)	12	52,776.70 (18,241.54)	12	69,680.86 (69,169.16)
Mild visual impairment (≥70–<80 letters)	23	61,665.78 (28,242.42)	23	82,772.30 (60,626.62)
No visual impairment (≥80 letters)	19	72,635.23 (31,806.99)	19	85,740.49 (73,439.94)

ETDRS, Early Treatment Diabetic Retinopathy Study; JPY, Japanese Yen; SD, standard deviation.

*Visual impairment is defined according to World Health Organization categories (WHO 2016) [28].

#### Secondary caregiver assessments

The mean (SD) CES-D scores were 10.59 (3.64) (overall), 10.40 (3.89) (caregivers of patients using T&E), 10.20 (4.32) (caregivers of patients using PRN), and 11.39 (2.66) (caregivers of patients who switched from PRN to T&E). Only 3 (4.2%) caregivers presented with a depressive mood (as indicated by a total CES-D score ≥16). There was no difference in the mean CES-D score for caregivers of patients with bilateral compared with unilateral wAMD (10.6 [3.97] vs. 10.5 [2.72]; p = 0.878). There were no clear associations between CES-D score and sex (patients or caregivers), caregiver status (primary caregiver or not), perceived stress for waiting time in hospital, and caregiver-perceived visual impairment (data not shown).

### Patient assessments

#### Resource use

The mean number of hospital visits for patients using T&E compared with PRN was 7.88 versus 14.0 (p = 0.00674) in year 1 and 5.68 versus 9.0 in year 2, respectively. For patients who switched from PRN to T&E, the mean number of hospital visits decreased from 13.21 to 7.43 (p<0.0001) in the first year after switch (Table 5). The mean number of injections for patients using T&E compared with PRN was 5.95 versus 3.40 (p = 0.000562) in year 1 and 4.09 versus 1.40 (p = 0.00183) in year 2. In patients who switched from PRN to T&E, there was no difference in the number of injections in year 1, but there were significantly fewer injections with PRN compared with T&E in year 2 (1.22 vs 4.62; p<0.0001) (Table 5).

**Table 5:** Resource use (hospital visits and number of injections) by treatment category in patients.

Year From Treatment Start	Treatment Regimen								
	PRN		T&E		P-value*	PRN to T&E Switchers**			
						n	PRN	T&E	P-value*
	n	Mean (SD)	n	Mean (SD)			Mean (SD)	Mean (SD)	
Number of hospital visits by year									
First year	10	14.00 (5.54)	41	7.88 (3.15)	0.00674	14	13.21 (4.66)	7.43 (3.76)	<0.0001
Second year	10	9.00 (5.56)	22	5.68 (1.99)	0.0961	9	6.33 (4.24)	5.23 (2.31)	0.2468
Number of injections by year									
First year	10	3.40 (1.58)	41	5.95 (1.24)	0.000562	14	3.86 (1.96)	4.50 (1.70)	0.1676
Second year	10	1.40 (2.01)	22	4.09 (0.87)	0.00183	9	1.22 (1.64)	4.62 (2.33)	<0.0001

PRN, as-needed; SD, standard deviation; T&E, treat-and-extend.

*PRN versus T&E, calculated by paired *t* tests.

**In patients who switched from PRN to T&E, assessments were made in patients who had 1 and 2 years of follow-up for both regimens.

#### Visual acuity outcomes

**Fig 3:**
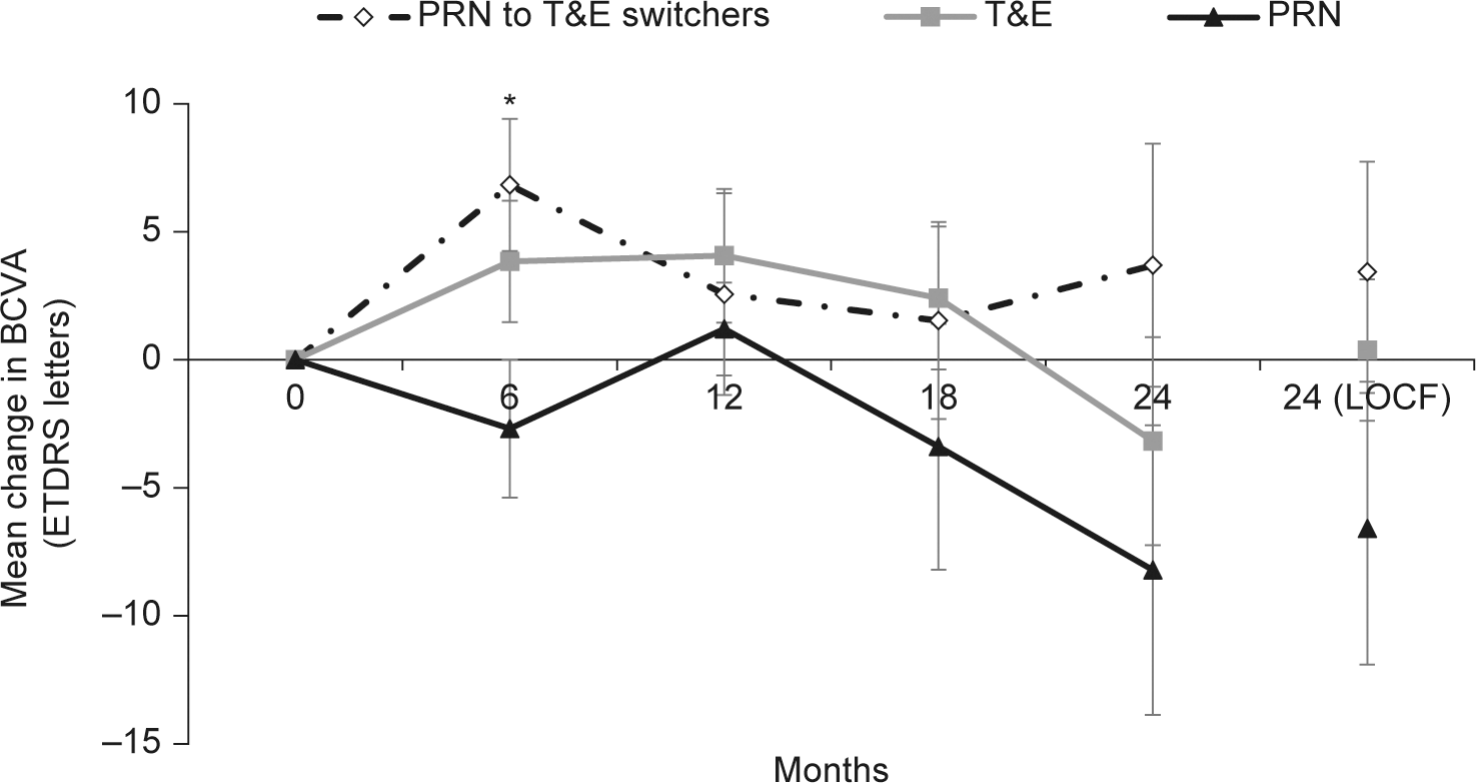
Mean change in BCVA (ETDRS letters) score over 2 years by treatment category (better-seeing eye). BCVA, best-corrected visual acuity; ETDRS, Early Treatment Diabetic Retinopathy Study; LOCF; last observation carried forward; PRN, as-needed; T&E, treat-and-extend. For time points 6, 12, 18, 24, and 24 (LOCF) months, respectively, n = 18, 16, 15, 16, and 18 (PRN to T&E switchers), n = 38, 33, 27, 22, and 39 (T&E), and n = 10, 10, 10, 9, and 10 (PRN). *p<0.05 versus baseline (paired *t* test).

At baseline, the BCVA (better-seeing eye) in the T&E, PRN, and PRN to T&E groups, respectively, were: severe visual impairment (1 patient, 0 patients, 0 patients); moderate visual impairment (9 patients, 0 patients, 3 patients); some visual impairment (7 patients, 2 patients, 4 patients); mild visual impairment (13 patients, 3 patients, 7 patients); no visual impairment (9 patients, 5 patients, 4 patients). Every patient (71 patients; 91 eyes) received anti-VEGF therapy for the treatment of wAMD, which included IVT-AFL (81 eyes; 89.0%), ranibizumab (7 eyes; 7.7%), and IVT-AFL/ranibizumab (3 eyes; 3.3%). No eyes received pegaptanib sodium or photodynamic therapy. A total of 42 eyes (46.2%) switched from ranibizumab to IVT-AFL. The mean ETDRS letter changes from baseline to year 1 were: +4.06 letters (T&E), +2.56 letters (patients who switched from PRN to T&E), and +1.20 letter (PRN) (Fig 3).

## Discussion

wAMD is associated with a high burden for both patients and caregivers. Previous studies have found that accompanying patients to hospital for treatment and/or monitoring is a high burden for caregivers [12,18]. In one study, patients with wAMD and caregivers from Japan visited the hospital frequently, but patients only received anti-VEGF treatment at 35% of these visits (data on file). We also showed that the majority of caregivers accompanied patients to the hospital every time, and 37% of caregiving time per year was spent on these visits. Not surprisingly, the costs associated with accompanying patients to hospital visits were high; this was based on an assumption that the clinic visit resulted in an entire day of not working/lost productivity, which is consistent with previous studies [12]. The majority of caregivers also waited during each visit and rated this time as stressful. We found that the ratio of injections to visits improved in patients receiving anti-VEGF T&E compared with PRN. The mean number of hospital visits was reduced by nearly half in patients who received T&E compared with PRN (7.88 vs 14.00 the first year), and was also significantly reduced when patients switched from PRN to T&E. As a result, T&E was associated with a reduction in caregiver-related burden, including costs and time. Although the total caregiver BIC-11 scores trended slightly higher among those caring for patients who switched from PRN to T&E compared with those who did not switch, this may be explained by the longer duration of disease and treatment history among the switch group; higher BIC-11 scores may have also been a driver for switching to T&E; however, these differences were not statistically significant. This study also provides useful information on the caregiver profile and their situation; most were female (75%), spouse/partner (55%), and nearly half (46%) were employed. The majority provided primary care (86%), and most drove to the hospital visit (80%), which was an average distance of 63 km. This study highlights the importance of examining ways to reduce the burden of visits *via* the use of home injections or satellite clinics.

wAMD is regarded as having a high burden on patients and caregivers; however, we showed that the caregiver BIC-11 scores were low. This may be due to successful treatment or to a number of additional factors, including the severity of visual impairment among the patients in our study population. Indeed, Braich et al. [29] showed a higher burden among caregivers of patients stratified into three groups according to degree of severe visual impairment (group 1, 20/200–10/200; group 2, 10/200 to light perception, and group 3, no light perception). BIC score was 7.00 for group 1, 8.66 for group 2, and 16.71 for group 3. As the baseline visual acuity for the population of the current study is better than that of the least severely affected patient group (group 1) in the study by Braich et al. [29], it is not surprising that we have shown a reduced burden in comparison. Interestingly, although there seems no association between actual visual acuity and BIC-11 total score in our study, when the data are stratified by the caregiver's perceived patient visual acuity, the more severe the perceived visual acuity, the higher the BIC-11 score—possibly indicating that burden may be partly explained by “perception.” In the study by Braich et al. [29], the prevalence of caregiver depression also increased with degree of visual impairment, from 16% in the 20/200 group to 48% in the no light perception cohort (p<0.01). In another study, Shima et al. [25] administered the CES-D to 224 healthy volunteers as a control group to patients with depression. The mean (SD) total score was 8.9 (7.1) points, and was significantly (p<0.05) higher for males (10 [6.9] points) than females (7.7 [7.1] points). Given that the majority of caregivers in our study are female, the CES-D scores may be biased compared with a normal population comprising males and females.

In a systematic review of eight studies using a T&E protocol and 62 studies using a PRN protocol, the mean improvement in visual acuity was 5.4 ETDRS letters in the PRN group and 10.4 ETDRS letters in the T&E group [30]. The mean number of anti-VEGF injections at 1 year was 5.60 (PRN) and 8.09 (T&E). Fewer injections were received by the respective PRN or T&E groups at 1 year in the current study, compared with the meta-analysis. The differences in injection number may be due to the treating physician’s decision to inject or not, which may also be guided by a patient’s preference to skip treatment if their eyes are dry or because of economic pressures, especially with the PRN regimen. Another possibility may be the 3-month extended injection interval following the loading phase that was used in patients with stable eye disease in the first year of the study. Subsequently, this regimen was changed to gradually increase the interval after the loading phase by 2-week iterations.

Although the study used validated questionnaires, it has a number of limitations inherent in an observational design. The study was performed in a single center in a rural area of Japan, where access to public transport was limited. The findings may not be reflective of wider practices, and generalizability of the study results is limited. The caregiver questionnaires were only administered once during the cross-sectional study, and this may provide limited information on the regimens over time. There was a discrepancy between the target and actual participant numbers, which was mainly the result of the lower than expected number of potential participants who met the inclusion criteria at the study site. Based on a small sample size, particularly in treatment switch groups, care must be taken when interpreting these findings.

## Conclusions

Treatment of wAMD with anti-VEGF agents via T&E rather than PRN reduced the number of hospital visits; this was associated with a reduction in caregiver burden, including time, costs, and emotional impact of accompanying patients to the hospital. It is also interesting to note that burden was associated with the caregiver’s perception of visual acuity rather than actual visual acuity; this may have implications for further studies aimed at assessing caregiver burden. This study may indicate the wider benefits associated with T&E regimens, including switching from an anti-VEGF PRN to a T&E regimen.

## Supporting information

S1 File Table A in S1 File. BIC-11 Item response distribution. Table B in S1 File. BIC-11 Total score stratified by caregiver age, sex, caregiver status, and perceived stress for waiting time.
(DOCX)
