# Supplementary material for: The impact of different anti-vascular endothelial growth factor treatment regimens on reducing burden for caregivers and patients with wet age-related macular degeneration in a single-center real-world Japanese setting
Source: PLoS One. 2017 Dec 8;12(12):e0189035. doi: 10.1371/journal.pone.0189035 (PMC5722328; doi:10.1371/journal.pone.0189035)
Supplement: S1 File — Table A in S1 File. BIC-11 Item response distribution. Table B in S1 File. BIC-11 Total score stratified by caregiver age, sex, caregiver status, and perceived stress for waiting time. (DOCX) [file pone.0189035.s001.docx]

**SUPPLEMENTAL DATA**

**Table A. BIC-11 Item Response Distribution.**

| **Item** | | **PRN to T&E switchers  (n = 18)** | **T&E  (n = 42)** | **PRN  (n = 10)** |
| --- | --- | --- | --- | --- |
|  |  | **Mean (SD)** | **Mean (SD)** | **Mean (SD)** |
| 1 | I do not have enough time for myself because of caregiving | 0.56 (1.10) | 0.64 (0.82) | 0.70 (0.48) |
| 2 | I cannot freely leave the house because of caregiving | 0.61 (1.20) | 0.57 (0.80) | 0.70 (0.48) |
| 3 | I am completely distressed by caregiving | 0.56 (1.04) | 0.36 (0.62) | 0.50 (0.71) |
| 4 | I want to delegate the care to someone else | 0.44 (0.78) | 0.43 (0.74) | 0.30 (0.48) |
| 5 | I am experiencing hardship because caregiving does not give me a sense of satisfaction | 0.61 (0.98) | 0.40 (0.66) | 0.40 (0.52) |
| 6 | Caregiving is hard because I cannot find the meaning of nursing | 0.61 (0.98) | 0.40 (0.66) | 0.40 (0.52) |
| 7 | My body aches when nursing | 0.28 (0.75) | 0.29 (0.55) | 0.20 (0.42) |
| 8 | I have ruined my health because of nursing | 0.33 (0.97) | 0.31 (0.52) | 0.20 (0.42) |
| 9 | It is a burden that public nursing care service personnel enter our house | 0.44 (1.04) | 0.21 (0.52) | 0.30 (0.48) |
| 10 | I have a hard time because patients resent receiving public nursing care service | 0.28 (0.75) | 0.19 (0.40) | 0.50 (0.71) |
| 11 | Total care burden | 0.61 (0.98) | 0.48 (0.63) | 0.40 (0.70) |

Item scores ranges from 0 to 4. Higher score indicates greater burden.

BIC-11, Burden Index of Caregivers-11; PRN, as needed; SD, standard deviation; T&E, treat-and-extend.

**Table B.** **BIC-11 Total Score Stratified by Caregiver Age, Sex, Caregiver Status, and Perceived Stress for Waiting Time.**

| **Category** | **Mean (SD)** | **p Value** |
| --- | --- | --- |
| Caregiver sex | | |
| Male (n = 18) | 3.33 (4.13) | 0.3691 |
| Female (n = 53) | 4.94 (7.14) |  |
| Caregiver status | | |
| Primary caregiver (n = 61) | 4.74 (6.85) | 0.5219 |
| Not primary caregiver (n = 10) | 3.30 (3.95) |  |
| Perceived stress for waiting time | | |
| Very (n = 8) | 6.63 (6.86) | 0.7521 |
| Moderately (n = 32) | 3.91 (4.82) |  |
| Slightly (n = 22) | 4.59 (5.46) |  |
| Not at all (n = 8) | 5.38 (13.23) |  |
|  | Pearson's correlation coefficient  *r^2^ (95% CI)* | |
| Age (n = 71) | 0.03 (–0.20 to 0.26) | |
| Number of hospital visits in previous 12 months (n = 70) | –0.12 (–0.35 to 0.12) | |

p Values were calculated by analysis of variance.

Total scores ranges from 0 to 44. Higher score indicates greater burden.

BIC-11, Burden Index of Caregivers-11; CI, confidence interval; SD, standard deviation.
